# Supplementary material for: Landscape Analysis of Public Health Jobs in India to Develop an Evidence-Based Public Health Curriculum
Source: Int J Environ Res Public Health. 2022 Nov 25;19(23):15724. doi: 10.3390/ijerph192315724 (PMC9738109; doi:10.3390/ijerph192315724)
Supplement: Supplementary file 1 [file ijerph-19-15724-s001.zip › ijerph-1996924-supplementary.pdf]

Table S1. STROBE Statement—Checklist of items that should be included in reports of ***cross-sectional studies***

	Item No	Recommendation	Page No
**Title and abstract**	1	*(a)* Indicate the study's design with a commonly used term in the title or the abstract<br>The study is a cross sectional study as stated on page1<br>Commonly used term: Public health jobs on page1	1
		(b) Provide in the abstract an informative and balanced summary of what was done and what was found<br>Provided in abstract on page1	1
**Introduction**			
Background/rationale	2	Explain the scientific background and rationale for the investigation being reported<br>Included in the Introduction on page 4.	4
Objectives	3	State specific objectives, including any prespecified hypotheses<br>Included on pages 4.	4
**Methods**			
Study design	4	Present key elements of study design early in the paper<br>Included in materials and methods section on page4	4
Setting	5	Describe the setting, locations, and relevant dates, including periods of recruitment, exposure, follow-up, and data collection<br>Included in materials and methods section on pages 4,5 and 6	4-6
Participants	6	*(a)* Give the eligibility criteria, and the sources and methods of selection of participants<br>Included in the materials and methods and results on pages 4-7	4-7
Variables	7	Clearly define all outcomes, exposures, predictors, potential confounders, and effect modifiers. Give diagnostic criteria, if applicable<br>Included in the materials and methods and results on pages 5, 6 and 7	5-7
Data sources/ measurement	8*	For each variable of interest, give sources of data and details of methods of assessment (measurement). Describe comparability of assessment methods if there is more than one group<br>Included in the materials and methods and results on pages 4-7	4-7
Bias	9	Describe any efforts to address potential sources of bias<br>Included in the materials and methods and results on pages 5	5
Study size	10	Explain how the study size was arrived at<br>Included in material methods and results section pages 4-7	4-7
Quantitative variables	11	Explain how quantitative variables were handled in the analyses. If applicable, describe which groupings were chosen and why<br>Included in material methods and results section page6	6
Statistical methods	12	*(a)* Describe all statistical methods, including those used to control for confounding<br>Included in material methods section page6	6
		(b) Describe any methods used to examine subgroups and interactions<br>Not applicable	
		(c) Explain how missing data were addressed<br>Not applicable	

		(d) If applicable, describe analytical methods taking account of sampling strategy<br>Not applicable	
		(e) Describe any sensitivity analyses<br>Not applicable	
**Results**			
Participants	13*	(a) Report numbers of individuals at each stage of study—eg numbers potentially eligible, examined for eligibility, confirmed eligible, included in the study, completing follow-up, and analysed<br>Included in results section pages6-7	6-7
		(b) Give reasons for non-participation at each stage<br>Included in results section pages6-7	6-7
		(c) Consider use of a flow diagram<br>Included in results section pages6-7	6-7
Descriptive data	14*	(a) Give characteristics of study participants (eg demographic, clinical, social) and information on exposures and potential confounders<br>Included in results section pages6-7	6-7
		(b) Indicate number of participants with missing data for each variable of interest<br>Included in results section pages6-7	6-7
Outcome data	15*	Report numbers of outcome events or summary measures<br>Included in results section pages 6-14	6-14
Main results	16	*(a)* Give unadjusted estimates and, if applicable, confounder-adjusted estimates and their precision (eg, 95% confidence interval). Make clear which confounders were adjusted for and why they were included<br>Included in results section pages 6-14	6-14
		(b) Report category boundaries when continuous variables were categorized<br>Not applicable	
		(c) If relevant, consider translating estimates of relative risk into absolute risk for a meaningful time period<br>Not applicable	
Other analyses	17	Report other analyses done—eg analyses of subgroups and interactions, and sensitivity analyses<br>Not applicable	
**Discussion**			
Key results	18	Summarise key results with reference to study objectives<br>Included in discussion section pages 13-15	13-15
Limitations	19	Discuss limitations of the study, taking into account sources of potential bias or imprecision. Discuss both direction and magnitude of any potential bias<br>Included in discussion section pages 15	15
Interpretation	20	Give a cautious overall interpretation of results considering objectives, limitations, multiplicity of analyses, results from similar studies, and other relevant evidence<br>Included in discussion section pages 14-16	14-16

Generalisability	21	Discuss the generalisability (external validity) of the study results<br>Included in discussion and conclusions section page15-16	15-16
**Other information**			
Funding	22	Give the source of funding and the role of the funders for the present study and, if applicable, for the original study on which the present article is based<br>Not applicable	

*Give information separately for exposed and unexposed groups.

Note: An Explanation and Elaboration article discusses each checklist item and gives methodological background and published examples of transparent reporting. The STROBE checklist is best used in conjunction with this article (freely available on the Web sites of PLoS Medicine at http://www.plosmedicine.org/, Annals of Internal Medicine at http://www.annals.org/, and Epidemiology at http://www.epidem.com/). Information on the STROBE Initiative is available at www.strobe-statement.org.
